# Genome-Wide Identification and Expression Analysis of the *ALDH* Gene Family in *Sinonovacula constricta* Bivalve in Response to Acute Hypersaline Stress

**DOI:** 10.3390/ani15010064

**Published:** 2024-12-30

**Authors:** Jianing Yu, Biao Wu, Yinghui Dong, Zhihua Lin, Hanhan Yao

**Affiliations:** 1 College of Advanced Agricultural Sciences, Zhejiang Wanli University, Ningbo 315101, China; yujianing134@163.com (J.Y.); dongyinghui@zwu.edu.cn (Y.D.); 2 Key Laboratory of Sustainable Development of Marine Fisheries, Ministry of Agriculture and Rural Affairs, Yellow Sea Fisheries Research Institute, Chinese Academy of Fishery Sciences, Qingdao 266071, China; wubiao@ysfri.ac.cn; 3 College of Marine Life Sciences, Ocean University of China, Qingdao 266003, China

**Keywords:** *Sinonovacula constricta*, ALDH gene family, osmotic pressure regulation, aldehyde dehydrogenase, oxidative stress, proline, salinity

## Abstract

The razor clam *Sinonovacula constricta*, a significant marine bivalve species, inhabits estuaries and encounters salinity stress. Despite its commercial importance, there is limited understanding of its adaptive mechanisms to high salinity. Aldehyde dehydrogenases (ALDHs), which belong to the NAD(P)^+^-dependent superfamily, play a crucial role in stress resilience by participating in catabolic and anabolic pathways, such as carnitine synthesis, glycolysis, and amino acid metabolism. This study presents the first comprehensive analysis of the ALDH family in *S. constricta* under acute high salt stress conditions and identifies 16 *ScALDH* genes across 10 subfamilies. These genes are located on eight chromosomes, with tandem duplications observed on chromosome 10; they encode mostly acidic and hydrophilic proteins. Among them, *ScALDH18A1* contains a conserved P5CS domain that is implicated in proline synthesis and osmotic regulation. The expression of 14 *ScALDH* members were significantly altered under acute salt stress conditions, with *ScALDH8* and *ScALDH18A1* showing increased expression levels, suggesting their involvement in osmotic pressure regulation. This research provides insights into the characteristics, evolution, and response to salinity stress of the *ScALDH* gene family while shedding light on ALDH function in bivalves, as well as serving as a foundation for further studies on osmotic stress regulation.

## Simple Summary

The razor clam *Sinonovacula constricta*, a commercially valuable marine bivalve species, is typically found in estuaries and coastal mudflat areas. However, limited research has been conducted on their adaptation mechanisms to high salinity stress. The present study provides the first comprehensive analysis of the members, gene structure, chromosome location, systematic evolution, and expression characteristics of the ALDH family in *S. constricta* following acute high salt stress. In this study, a total of 16 *ScALDH* genes were identified. Under acute salt stress, the expression of 14 members of the ScALDH family significantly changed, compared with when they were at 0 h, suggesting that the *ScALDH* gene family plays a crucial role in facilitating the regulation of osmotic pressure under salinity stress and adaptation. This study elucidated the gene characteristics, systematic evolution, and response to acute high salt stress of the *ScALDH* family, contributing valuable data to the understanding of the ALDH gene family in bivalves and providing a reference for further exploration into their role in osmotic stress regulation.

## 1. Introduction

The razor clam *Sinonovacula constricta*, one of the four traditional cultured shellfish in China, primarily inhabits estuaries and coastal mudflat areas [1]. In recent years, climate change factors, such as high temperature, drought, typhoon, etc., have led to significant fluctuations in sea water salinity, especially in coastal mudflats, estuaries, and intertidal zones [2]. Salinity plays a crucial role in constraining the natural distribution and artificial cultivation of marine shellfish, exerting profound effects on their physiology, biochemistry, and osmoregulation, as well as growth and metabolism [3]. Throughout the course of long-term evolution, aquatic organisms have developed adaptive mechanisms to effectively cope with salinity fluctuations and maintain an optimal metabolic state [4]. Studies have demonstrated that razor clam resistance to salinity stress is mediated by diverse biological processes encompassing behavioral adaptations, osmoregulation, and immune responses [5].

Under conditions of drought, salinity, dehydration, and other environmental stresses, organisms exhibit excessive endogenous aldehyde molecule production, leading to the disruption of cellular homeostasis, enzyme inactivation, and cell death [6,7,8]. Therefore, maintaining a balanced level of aldehyde within the body is crucial for ensuring proper cellular growth and development. Aldehyde dehydrogenase (ALDH) is an NAD(P)^+^-dependent gene superfamily that catalyzes the irreversible conversion of various active aldehydes into non-toxic carboxylic acids [9]. Currently, 24 families of ALDH have been identified, with 14 families found in plants and seven being plant-specific; the human ALDH family comprises more than 19 members [10,11]. In aquatic organisms, recent studies have demonstrated that environmental stresses such as hypoxia, salinity, or high temperature can activate *ALDH* genes [12,13,14]. It was shown that the spatial distribution of *ALDH* in crucian carp *Carassius carassius* is very different from the conserved distribution in other vertebrates, which may be related to the fact that acetaldehyde can competitively inhibit the oxidation of other endogenous aldehydes in crucian carp under hypoxic conditions [14]. Aldehyde dehydrogenase prevents the accumulation of toxic aldehyde compounds, which in the blue mussel *Mytilus galloprovincialis*, European green crab *Carcinus maenas*, and Asian clam *Corbicula fluminea* [15,16,17]. However, this gene family has not yet been well investigated in mollusks.

The most remarkable among ALDHs is the ALDH18 family, which belongs to a distant bifunctional enzyme group within the ALDH superfamily. The encoded protein plays a crucial role in proline biosynthesis by catalyzing the reduction of glutamate to delta1-pyrroline-5-carboxylate, making this family rate-limiting enzymes for proline synthesis [18,19]. Proline (Pro), as one of the major free amino acids (FAAs), accumulates rapidly in organisms under increased salinity. The synthesis and metabolism of Pro help the mitochondria alleviate oxidative stress and provide additional energy for cells experiencing hyperosmolarity, thereby slowing down cell damage and cell turnover, maintaining cell integrity, and ultimately enhancing salt tolerance in mollusks [20,21].

In this study, we conducted a comprehensive genome-wide identification of the ALDH superfamily in *S. constricta* (*ScALDH*), analyzing their conserved structural domains, motifs, phylogenetic relationships, and expression characteristics under salinity stress. These findings laid a theoretical foundation for further exploration into the function and regulatory mechanisms of ALDH in mollusks.

## 2. Materials and Methods

### 2.1. Experimental Animals

In June 2024, 500 razor clams were collected from Dingzi Bay on the Shandong Peninsula in China (120°83′ N, 36°60′ E, average water salinity 30.0 ± 1 ppt), with an average shell length of 51.31 ± 2.07 mm and a body weight of 9.34 ± 1.72 g. Prior to the commencement of the experiment, the razor clams were acclimatized in seawater at a water temperature of 20 ± 1 °C and salinity of 30 ± 1 ppt for a duration of five days. Sea crystals (Jiangsu Yandao Logistics Co., Ltd., Jintan, Changzhou, China) were used to salinize the water to the required salinity. During this period, daily water changes were performed, while *Chlorella vulgaris* was provided as feed both in the morning and evening.

### 2.2. Expression Analysis of ScALDHs Under High Salt Stress

The mature razor clams, exhibiting good vitality, precise specification, and intact shells, were selected as the experimental materials. Healthy razor clams were randomly selected for acute salinity stress, with a control group (30 ppt) and a high-salinity group (40 ppt), each comprising three biological replicates. Within each group, three clams were chosen, and gill tissues at 0 h, 6 h, and 12 h post-salinity stress were dissected, rapidly frozen in liquid nitrogen, and subsequently stored at −80 °C for RNA extraction.

### 2.3. RNA Extraction and qRT-PCR

The total RNA from tissues was extracted using Trizol, followed by the examination of RNA degradation and contamination on a 1% agarose gel, and then the concentration of extracted RNA was determined using a microspectrophotometer (NanoPhotometer^TM^, Munich, Germany). The PrimeScript RT kit was employed for cDNA synthesis, and the resultant cDNA was stored at −20 °C for subsequent experiment. The primers were designed using the Primer 3 online website (Table 1), and *RS9* (40S ribosomal protein S9) was selected as the internal reference gene. The qRT-PCR reaction system (10 μL) consisted of 0.2 μL each of upstream and downstream primers, 1 μL of template cDNA (1000 ng/μL), 5 μL of 2 × ChamQ SYBR Color qPCR Master mix, and 3.6 μL of DEPC water. The reaction procedure involved an initial denaturation at 95 °C for 30 s, followed by amplification with 40 cycles of denaturation at 95 °C for 10 s, and annealing at 60 °C for 30 s, and the melting curve analysis was performed at 95 °C for 15 s and 60 °C for 60 s. All samples were analyzed in triplicate both biologically and technically.

### 2.4. Identification and Gene Structure Analysis of ScALDHs

The ALDH amino acid sequences of *Homo sapiens* (GCF_000001405.40) and *Drosophila melanogaster* (GCF_000001215.4) were downloaded from the NCBI database (https://www.ncbi.nlm.nih.gov/, accessed on 13 July 2024) as reference sequences, and subsequently, the TBtools software was employed to perform BLAST alignment in order to identify homologous sequences in the genome of *S. constricta* (WSYO00000000.1), with a significance threshold set at an e-value of 1 × 10^−5^. The Hidden Markov Model of the Pfam ALDH domain (PF00171) was obtained from the Pfam database (http://pfam-legacy.xfam.org/, accessed on 15 July 2024) and subsequently compared to the constrictor proteome using the HMM Search functionality within the TBtools software. Gene structure analysis was performed using TBtools software (v1.0987663). NCBI CD-search (https://www.ncbi.nlm.nih.gov/Structure/cdd/wrpsb.cgi, accessed on 15 July 2024) was employed to search for conserved structural domains of *ScALDHs*, and combined with the literature, the members of the *ScALDH* gene superfamily were selected. The protein physicochemical properties of *ScALDHs* were predicted using the ExPAsy online software (https://www.expasy.org/, accessed on 15 July 2024), which included the determination of molecular weight (MW), isoelectric point (pl), instability index (II), and grand average of hydropathicity (GRAVY). Subcellular localization prediction was performed using Cell-PLoc 2.0 (http://www.csbio.sjtu.edu.cn/bioinf/Cell-PLoc-2/, accessed on 16 July 2024) and WoLF PSPRT (https://wolfpsort.hgc.jp/, accessed on 16 July 2024). The member domains of identified *ScALDHs* were analyzed using the NCBI-CDD database (https://www.ncbi.nlm.nih.gov/cdd/, accessed on 22 July 2024), and the conserved domain of the *ScALDHs* protein was predicted by combining SMART online website (https://smart.embl.de/, accessed on 23 July 2024) and Pfam. The gene structure was also predicted using the Visualize Gene Structure function of TBtools, and the schematic mapping of gene structure was generated through the online website GSDS (https://gsds.gao-lab.org/Gsds_help.php, accessed on 28 July 2024). Signal peptide prediction for *ScALDHs* was conducted using SignalP 4.1 (https://services.healthtech.dtu.dk/services/SignalP-4.1/, accessed on 2 August 2024).

### 2.5. Multiple Sequence Alignment and Phylogenetic Analysis

The genomic data of model animals and other mollusks were retrieved from the NCBI database, and their ALDH amino acid sequences were subjected to screening. The amino acid sequences of *ScALDHs* were aligned using the DNAMAN 6.0.3.99 software. The ALDH phylogenetic tree was constructed using the NJ method with 1000 bootstrap replicates in MEGA 11 software.

### 2.6. Motif Analysis and Chromosome Location of ScALDHs

Conserved motifs in *ScALDHs* were predicted based on amino acid sequences of the *ScALDH* superfamily using the MEME online website (https://meme-suite.org/meme/, accessed on 20 August 2024). The TBtools software was employed to map the CDS sequence of *ScALDHs* to the genome for chromatin localization and visualization, as described by Chen et al. [22].

### 2.7. Statistical Analyses

The relative expression of ScALDHs was determined using the 2^−ΔΔCt^ method. Statistical analysis was performed using SPSS (v26) with one-way ANOVA, where *p* < 0.05 indicated significance, and *p* < 0.01 indicated high significance. Graphical representation of the expression data was generated using GraphPad Prism (v8.0.2) software.

## 3. Results

### 3.1. Gene Identification and Protein Characterization of ScALDHs

A total of 16 *ScALDHs* were retrieved from the *S. constricta* genome (Table 2), which were further classified into 10 subfamilies based on their sequence homology: *ScALDH1* (including *ScALDH1A-1*, *ScALDH1A-2*, *ScALDH1A-3*, *ScALDH1A-4*, *ScALDH1A-5*, *ScALDH1A-6*, and *ScALDH1L*), *ScALDH3*, *ScALDH4*, *ScALDH5* (*ScALDH5A-1* and *ScALDH5A-2*), *ScALDH6*, *ScALDH7*, *ScALDH8*, *ScALDH9*, and *ScALDH18* (*ScALDH18A1*). The amino acid composition of the *ScALDH* gene family members ranged from 138 to 921, with molecular weight ranging from 15.230 to 101.399 kDa. Most gene family members exhibited minimal variation in the number of encoded amino acids, with *ScALDH1L* encoding the longest sequence at 921 aa and *ScALDH5A-1* having the shortest at 138 aa. The isoelectric point fell within a range of 5.27 to 8.23, and all members were predominantly acidic proteins, except for *ScALDH5A-2*, *ScALDH3*, *ScADH6*, and *ScALDH7*, which display basic protein characteristics. Computational analysis showed that all members of the ScALDH protein family exhibited varying degrees of hydrophilicity, except *ScALDH5A-1*, *ScALDH5A-2*, and *ScALDH18A1*, which displayed hydrophobic characteristics. Subcellular localization analysis showed that *ScALDHs* were mainly located in the cytoplasm and mitochondria. Furthermore, signal peptide prediction indicated the absence of any signal peptides in members of the *ScALDH* gene family, suggesting the absence of secreted proteins.

### 3.2. Chromosome Localization Analysis

**Figure 1:**
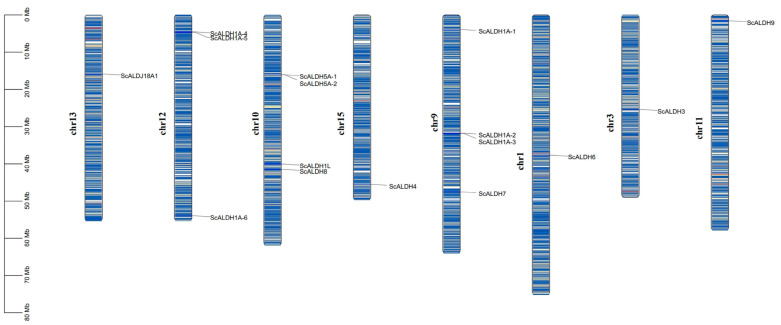
Chromosome location diagram of the *ScALDH* gene family in *S. constricta*.

The chromosomal locations of all members of the *ScALDH* gene family are shown in Figure 1, illustrating the localization of the 16 *ScALDH* genes across eight chromosomes. Notably, *ScALDH1* family members exhibited a higher abundance and were situated on chromosomes 9, 10, and 12. Additionally, *ScALDH3* was positioned on chromosome 3, while *ScALDH4* resided on chromosome 15. Chromosome 10 harbored both *ScALDH5* and *ScALDH8* genes, and tandem replication may occur within the *ScALDH5* family. Furthermore, chromosome 1 accommodated *ScALDH6*, whereas chromosome 9 housed *ScALDH7*. Lastly, *ScALDH9* and *ScALDH18* were observed on chromosome 11 and chromosome 13, respectively.

### 3.3. Multiple Sequence Alignment and Phylogenetic Analysis

**Figure 2:**
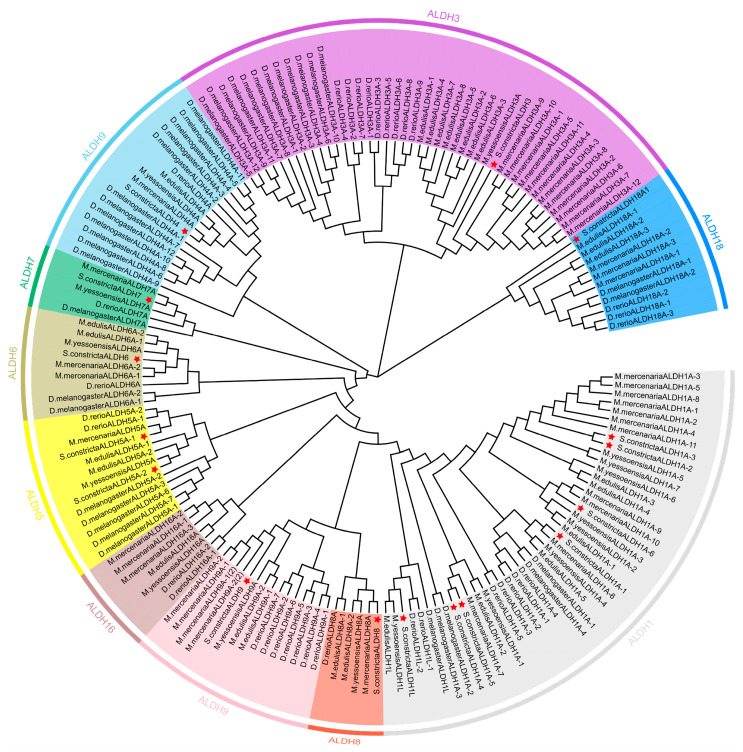
Phylogenetic tree of *ScALDH* gene family members among six species. The color of the branches represents the same gene family. The red star represents the *ALDHs* gene family of *S. constricta*. Abbreviations: *Drosophila melanogaster*: *D. melanogaster*; *Mercenaria mercenaria*: *M. mercenaria*; *Mizuhopecten yessoensis*: *M. yessoensis*; *Danio rerio*: *D. rerio*; *Mytilus edulis*: *M. edulis*; *Sinonovacula constricta*: *S. constricta*.

A total of 171 *ALDH* amino acids of *D. melanogaster*, *Mercenaria mercenaria*, *Mizuhopecten yessoensis*, *Danio rerio*, *Mytilus edulis*, and *S. constricta* were selected for multiple sequence alignment and phylogenetic analysis (Supplementary Figure S1, Figure 2). The multi-sequence alignment revealed a 32.68% similarity between the *ALDHs*, and the phylogenetic tree revealed the polymerization of these genes into three distinct evolutionary branches. *ScALDH3* and *ScALDH18* were found to be closely associated within one cluster, while *ScALDH1*, *ScALDH4*, *ScALDH5*, *ScALDH6*, *ScALDH7*, *ScALDH8*, *ScALDH9*, and *ScALDH16* formed a separate branch. Notably, the families of both *ScALDH8* and *ScALDH9* exhibited close relatedness within this branch. Furthermore, the *ScALDH3* and *ScALDH18* families were observed to form a distant cluster from the other gene families. The genes of the six species were initially clustered with the members from the same subfamily, followed by clustering with other subfamilies, indicating a high degree of conservation within the *ScALDH* superfamily. All *ScALDH* genes clustered closely with similar genes from other known species, primarily aligning with hard clams *M. mercenaria*, then aligning closer to *M. edulis* and *M. yessoensis*, next to *D. rerio*, and furthest away from *D. melanogaster*, which is consistent with the traditional taxonomic classification.

### 3.4. Analysis of Gene Structure Characteristics

**Figure 3:**
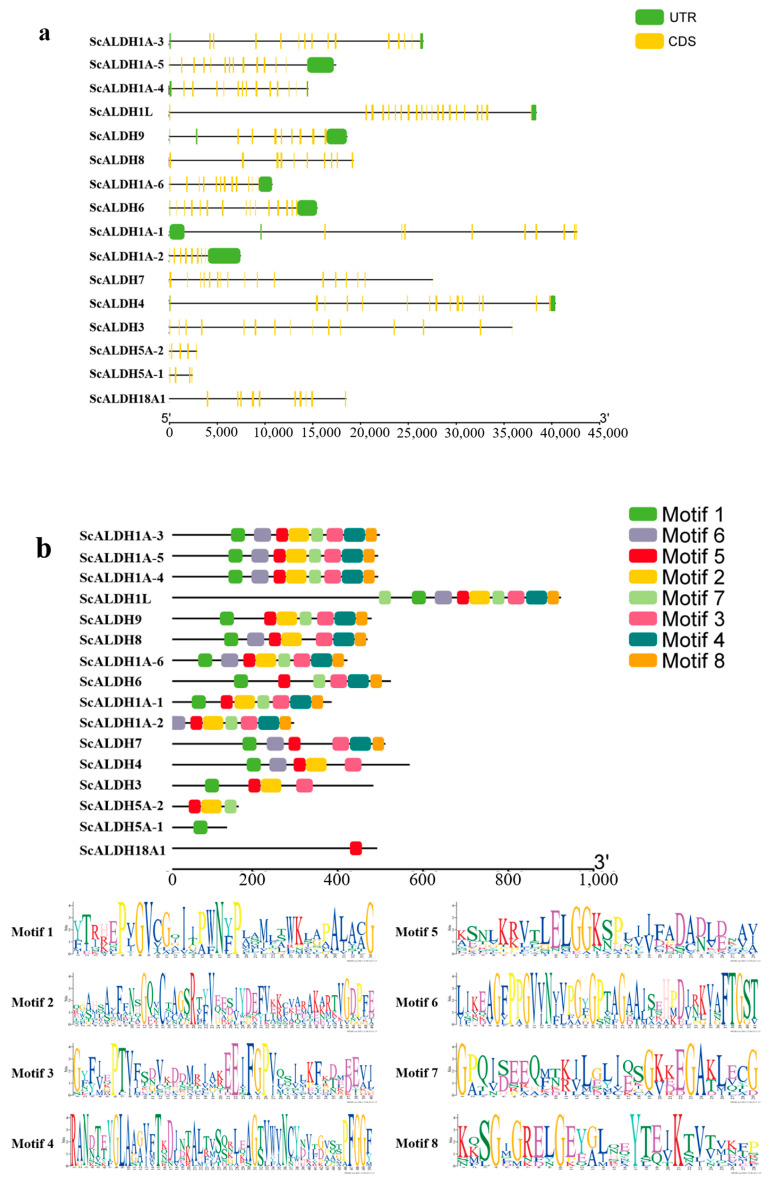

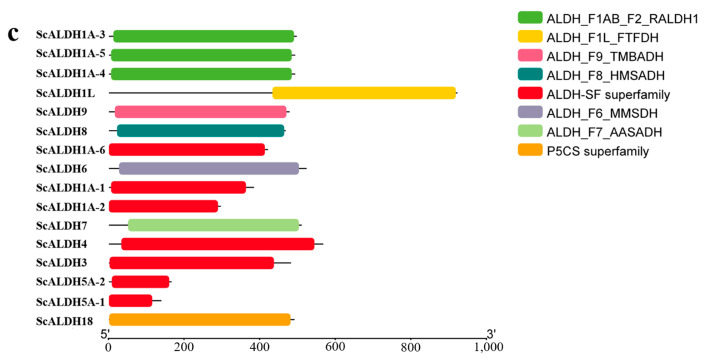
Schematic diagrams of the domains of the *ScALDH* gene family. (**a**) Genetic structure diagram of *ScALDHs*; (**b**) the conserved motif distributions of *ScALDHs*; (**c**) conservative domain analysis of *ScALDHs*.

The gene structures of the 16 members belonging to the *ScALDH* family are shown in Figure 3a. There were variations in both the length and coding region among different genes, with *ScALDH1A-1* being the longest and *ScALDH5A-1* being the shortest. Analysis of the conserved motifs in the *ScALDH* superfamily proteins (Figure 3b) revealed significant variability, although the majority of *ScALDH* motifs within the same subgroup exhibited similarities in both composition and sequence. The *ScALDH1* family exhibited the highest degree of conservation, characterized by the presence of eight conserved motifs. Conversely, *ScALDH18* solely possessed motif 5, while *ScALDH5A-1* was limited to motif 1, due to its relatively short protein sequence. Furthermore, it is noteworthy that a majority of the family members exhibited the occurrence of motif 1, motif 2, motif 6, motif 5, and motif 3.

Structural domain prediction results revealed (Figure 3c) that *ScALDH1* families encompassed ALDH-F1AB-F2-RALDH1 and ALDH-SF superfamily domains; the *ScALDH3*, *ScALDH4*, and *ScALDH5* families all possessed the ALDH-SF superfamily domain, whereas only *ScALDH18* exhibited conserved P5CS superfamily domains.

### 3.5. Analysis of Gene Expression Patterns Under Acute Hypersaline Stress

**Figure 4:**
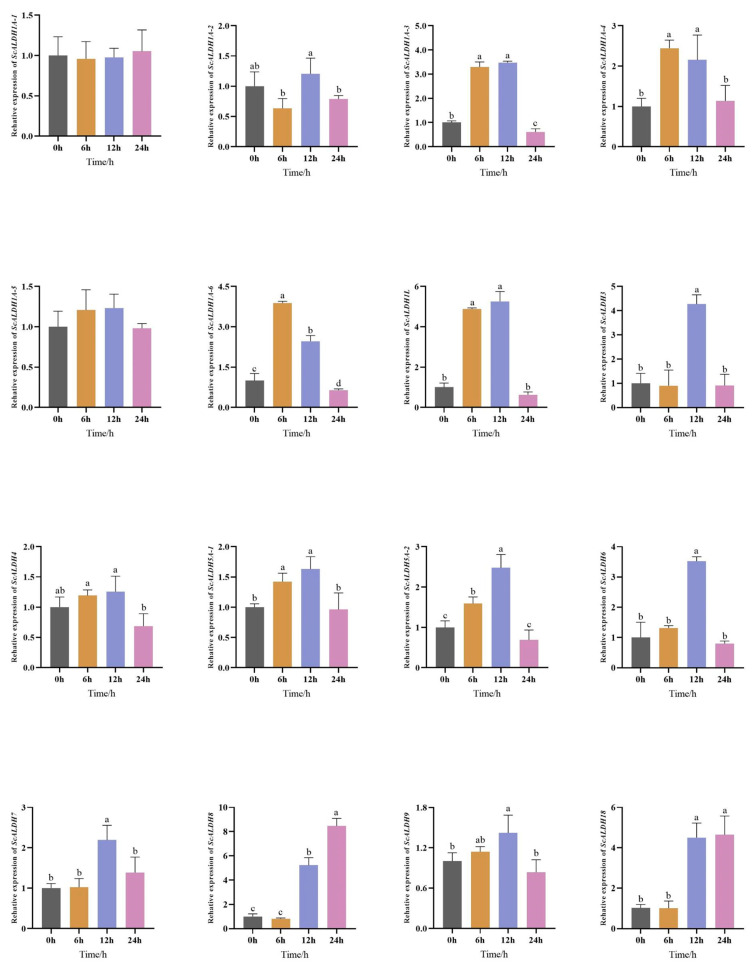
Relative expression levels of ALDH gene family members in gill tissues of *S. constricta* (n = 4). Different letters indicate significant differences (*p* < 0.05) among different genes.

The members of the ALDH superfamily in the gill tissue of razor clams at a salinity of 40 were quantitatively analyzed, and the results are shown in Figure 4. All *ScALDH* genes were expressed in gill tissues, with significant changes (*p* < 0.05) in 14 genes after acute high salt stress, except for *ScALDH1A-1* and *ScALDH1A-5*. Compared to the initial time point (0 h), the expression of *ScALDH1A-2* initially increased and then decreased, reaching its peak at 12 h. Similarly, the expression levels of *ScALDH1A-3*~*ScALDH7* and *ScALDH9* exhibited an initial increase followed by a decrease, peaking at either 6 h or 12 h before returning to baseline gene expression levels at 24 h. Notably, both *ScALDH8* and members of the *ScALDH18A1* family showed a consistent increasing trend throughout the entire duration, reaching their highest levels at 24 h. The difference between expressions at 12 h and 24 h compared to that at time point 0 h was highly significant.

## 4. Discussion

Aldehyde dehydrogenase superfamily proteins are present in various subcellular compartments, including the mitochondria, cytoplasm, endoplasmic reticulum, and the nucleus. Organisms are exposed to oxidative stress and the consequent generation of reactive oxygen species (ROS). In animals, oxidants are produced during mitochondrial respiration, biological metabolism, and other processes leading to ROS formation, and ALDHs can alleviate oxidative stress by reducing aldehyde-induced damage [23]. The subcellular localization of this study indicates that *ScALDHs* predominantly localize in the cytoplasm and mitochondria. Therefore, it is speculated that ALDHs may indirectly mitigate cell ROS or reduce lipid peroxidation in the mitochondria to protect cells against diverse abiotic stresses [24,25,26].

ALDHs are NAD(P)^+^-dependent enzymes, and their presence in almost all genomes analyzed to date highlights their significance in biological function. As of July 2003, a total of 555 genes encoding the ALDH protein were identified, with 32 found in archaea, 351 in eubacteria, and 172 in eukaryotes [27]. In the human genome, there have been reports of 17 functional genes and 3 pseudogenes related to the ALDH [28]. The study of the ALDH superfamily has extensively focused on plants but remains unexplored in mollusks. Therefore, this research aimed to analyze the *S. constricta* ALDH family using the bioinformatics method for the first time. The results revealed a total of 16 genes within the ALDH gene family: seven *ScALDH1* members; one *ScALDH3* member; one *ScALDH4* member; two *ScALDH5* members; one *ScALDH6* member; one *ScALDH7* member; one *ScALDH8* member; one *ScALDH9* member; and one *ScALDH18* member. Throughout evolution, eukaryotes have undergone frequent replication events, including whole genome replication, as well as tandem replication, fragment replication, and transposable replication [29]. In this study, the *ScALDH5* family genes on chromosome 10 of razor clams were duplicated in series, and the expression level of *ScALDH5* family genes significantly changed after exposure to high salt stress conditions. These findings suggest that the duplication of *ScALDH-5* may better facilitate razor clams’ adaptation to changing environments [30,31].

ALDHs play crucial roles in various catabolic and biosynthetic pathways, encompassing carnitine biosynthesis, glycolysis/gluconeogenesis, and amino acid metabolism [32,33]. Upregulation of ALDH has been observed in numerous plant species exposed to heat, dehydration, salinity, oxidants, ultraviolet (UV) radiation, insecticides, or metals [34]. In aquatic animals, ALDH plays an important role in amino acid and flavor regulation in crucian carp *Eriocheir sinensis*, suggesting that it could be a candidate target gene to enhance the eating quality of hepatopancreases during the maturation of female carps, thereby increasing nutrient requirements and flavor production during the maturation stage [35]. *ALDH-E2* enhances free amino acid metabolism in razor clams under high salt conditions and may play an important role in regulating osmolality and flavor in razor clams [13]. The ALDH5 gene family comprises succinate semialdehyde dehydrogenase (SSADHs; EC 1.2.1.24), which catalyzes the conversion of succinate semialdehyde (SSA) to succinic acid during the final step of γ-aminobutyrate (GABA) catabolism. ALDH5 is involved in the GABA “shunt” pathway in bacteria, plants, and animals, enabling these organisms to bypass the tricarboxylic acid pathway. GABA plays a crucial role as a neurotransmitter in mammals. For instance, GABA has been shown to enhance the growth performance of juvenile *Oreochromis nilotica* and increase serum total protein activity and superoxide dismutase [36]. ALDH inhibits the replication of white spot syndrome virus (WSSV) at high temperatures, thereby reducing the mortality of infected shrimps, which is important in shrimp culture [12]. Members of the ALDH6 gene family are also referred to as methyl malondialdehyde (MMS) dehydrogenase (EC1.2.1.27), which facilitate reactions associated with valine and pyrimidine catabolism. Studies have demonstrated that the ALDH6 enzyme can metabolize malonic semialdehyde into acetyl coenzyme A [37]. The ALDH7 family members (EC 1.2.1.31) are known as ^∆^1-piperideine-6-carboxylate dehydrogenases (P6CDH), α-aminoadipic semialdehyde dehydrogenases, or antiquitins. The ALDH7 gene family exhibits high conservation across evolutionarily distant species, suggesting potential conservation of physiological functions as well. Upregulation of ALDH7B occurs in response to various stressors, such as ultraviolet radiation, dehydration, elevated salinity, hypothermia, heat shock, and ABA treatment [38,39]. In this study, we observed an initial increase followed by a decrease in the expression levels of most members of the *ScALDH5*, *ScALDH6*, and *ScALDH7* families and under high salt stress conditions, indicating their activation for removing excessive aldehydes and toxins while protecting organisms from damage caused by salinity stress. It is speculated that razor clams necessitate optimal enzymatic activity to effectively regulate and adapt to osmotic pressure fluctuations induced by salinity, thereby achieving organismal osmotic equilibrium over time. Furthermore, considering the highly conserved nature of this gene family, it is speculated that its pivotal role in promoting *S. constricta* growth and facilitating adaptation to environmental stress cannot be overlooked.

ALDH18, also known as P5CS, is a bifunctional enzyme comprising an N-terminal amino acid kinase domain and a C-terminal aldehyde dehydrogenase domain. This enzyme plays a crucial role in aldehyde metabolism and exhibits diverse metabolic functions in vivo. In mammals, ALDH18A1 catalyzes the ATP- and NADPH-dependent conversion of glutamic acid into pyrrolinr-5-carboxylic acid (P5C). P5C is subsequently converted to ornithine, which is then utilized in the de novo biosynthesis of proline and arginine [33]. Under conditions of water scarcity and salt stress, terrestrial plants exhibit significant up-regulation of ALDH18 expression [19].

The human ALDH18A1 gene encodes a bifunctional enzyme known as delta 1-pyrroline-5-carboxylate synthase (P5CS), which is responsible for catalyzing the initial two steps of proline, ornithine, and arginine biosynthesis [40,41]. In vertebrates, there exist two subtypes of ALDH18A1 genes that play a crucial role in regulating proline and ornithine biosynthesis in vivo [42,43]. It has been observed that salinity stress significantly enriches alanine, proline, and glutamate metabolism and other amino acid metabolic pathways. This finding suggests that free amino acids may have an important regulatory function in maintaining the osmotic pressure of *S. constricta* [44]. Similar to betaine, proline is a prevalent and compatible osmotic solute in plants, enabling the accumulation of proline to counterbalance the osmotic imbalance induced by water stress. This phenomenon holds true not only in higher plants but also in bacteria, protozoa, marine invertebrates, and algae [45,46]. In eukaryotes, proline synthesis occurs through the conversion of glutamate into D1-pyrroline-5-carboxylic acid (P5C), catalyzed by D1-pyrroline-5-carboxylic acid synthase (P5CS). P5CS functions as a bifunctional enzyme encompassing γ-glutamyl kinase (GK; EC 2.7.2.11) and glutamyl phosphate reductase (GPR; EC 1.2.1.41) activities found in prokaryotes [46]. As a rate-limiting enzyme in proline synthesis, the expression level of P5CS influences proline content. Conversely, *S. constricta* exhibited increased expression of the P5CS gene with prolonged salt stress, resulting in an elevated proline level under high salinity conditions, supporting its crucial role in osmotic pressure regulation [21]. In this study, the domain prediction results revealed that the *ScALDH18A1* gene possessed a conserved P5CS superfamily domain. Furthermore, its expression exhibited an upward trend under high salt stress, with a significant difference observed between 12 h and 24 h, compared to 0 h. These findings are consistent with previous studies and suggest that *ScALDH18A1*, as a rate-limiting enzyme in proline synthesis, plays a crucial role in enhancing resistance to harsh environments and protecting damaged tissues. Therefore, it is of great importance for osmotic regulation in razor clams.

In conjunction with the bioinformatics analysis findings of the aforementioned *ScALDH* family members, this study proposed a significant role for the *ScALDH* family in osmotic regulation of razor clams, thereby providing crucial insights for further investigations into the salt adaptation mechanism of *S. constricta*.

## 5. Conclusions

A total of 16 constrictor ALDH family members (*ScALDH1*, *ScALDH3*, *ScALDH4*, *ScALDH5*, *ScALDH6*, *ScALDH7*, *ScALDH8*, *ScALDH9*, *ScALDH18*) were identified in this study. Under acute high salt stress, the expression levels of 14 *ScALDH* family members exhibited significant changes, which preliminarily suggests that the ALDH family is related to osmotic stress regulation in razor clams. Overall, these findings provide a theoretical basis for further research on the osmotic pressure regulation mechanism of ALDH in mollusks.

## Figures and Tables

**Table 1:** The primer sequences of *ScALDH*.

Primer Name	Sequence (5′-3′)
*ScALDH1A-1*	F: TCAAGGAGGTGGGCAAGATT R: ACTTTCCTCCCAACTCCAGG
*ScALDH1A-2*	F: CGGCCTTGACCCTTAGTAGT R: TCCAATCCCCGACTGTTTGA
*ScALDH1A-3*	F: TGGGCGGACAAAATCTCTG R: GGGGCAATCTTCCAAGCAAA
*ScALDH1A-4*	F: GCTGATGCCAACCTTGAACT R: GCACATGGTCACAAACTCGT
*ScALDH1A-5*	F: GAAGAGGTCCTGGAGAGAGC R: AGTTGATCCAGACACTGCCA
*ScALDH1A-6*	F: AGCCTCCCTCATTCATCAGG R: GATCAGCTTGCCAACCTCTG
*ScALDH1L*	F: AATCACCGAGCACATCTGGA R: TGGGCTCCATGAACAATCCT
*ScALDH3*	F: CTGGCCAGACTTATCCCACA R: CAACCATTGAGTTCCCCGTG
*ScALDH4*	F: GATACAATCACGTCCGCACC R: CCGCCACATTCTCCAATCAG
*ScALDH5A-1*	F: AAGTCATGTATGGGGCGTCA R: TTTTGTTGGCATGGGAGTGG
*ScALDH5A-2*	F: GCCATTGATGCTCAGCTGAA R: AATGTGGCTCCCTTGACTCA
*ScALDH6*	F: TGCCCATGTTCTCCTTCACT R: TGGTGATGTAAGGGTGGCAT
*ScALDH7*	F: AGCACCCACTACACCATTGA R: AACCCAATATCAGCCCCTCC
*ScALDH8*	F: GGTTACTTCATGCGTCCCAC R: GACACTGTTTGCTCGCTTGA
*ScALDH9*	F: GGTATCGGGGCATGGAACTA R: TCTCCCAACATCACTGCAGT
*ScALDH* *18*	F: GCATTTACACAAGCCCACCA R: TTACCCGCAGCAACCTTAGA
*RS9*	F: TGAAGTCTGGCGTGTCAAGT R: CGTCTCAAAAGGGCATTACC

**Table 2:** Protein composition and physicochemical properties of the *ALDH* gene family in *S. constricta*.

Gene ID	Gene Name	Localization	Signal Peptide	Protein Length/aa	Molecular Weight (kD)	Isoelectric Point (pI)	Instability Index	Hydrophilicity
cds.evm.model.ctg396.33	*ALDH1A-3*	Cyto ^a^, Cyto ^b^, Mito ^b^	/	496	54.098	6.30	34.83	−0.209
cds.evm.model.ctg1159.6	*ALDH1A-5*	Cyto ^a^, Cyto ^b^, Mito ^b^	/	492	53.650	5.42	29.83	−0.184
cds.evm.model.ctg2688.1	*ALDH1A-4*	Cyto ^a^, Cyto ^b^, Mito ^b^	/	492	53.650	5.42	29.83	−0.184
cds.evm.model.ctg154.26	*ALDH1L*	Mito ^a^, Cyto ^b^	/	921	101.399	6.35	34.77	−0.150
cds.evm.model.ctg1336.8	*ALDH9*	Cyto ^a^, Cyto ^b^	/	477	51.315	5.28	33.34	−0.059
cds.evm.model.ctg848.1	*ALDH8*	Cyto ^a^, Cyto ^b^	/	467	50.838	6.72	31.03	0.019
cds.evm.model.ctg473.4	*ALDH1A-6*	Cyto ^a^, Cyto ^b^, Mito ^b^	/	420	45.335	5.42	22.67	−0.030
cds.evm.model.ctg267.5	*ALDH6*	Mito ^a^, Mito ^b^	/	522	56.976	8.23	24.45	−0.155
cds.evm.model.ctg807.23	*ALDH1A-1*	Cyto ^a^, Cyto ^b^	/	383	42.567	5.27	32.51	−0.125
cds.evm.model.ctg396.32	*ALDH1A-2*	Cyto ^a^, Cyto ^b^, Mito ^b^	/	295	31.907	6.55	36.78	−0.154
cds.evm.model.ctg85.30	*ALDH7*	Cyto ^a^, Mito ^b^	/	509	55.191	7.54	29.74	−0.063
cds.evm.model.ctg1032.7	*ALDH4*	Mito ^a^, Cyto ^b^	/	566	63.191	6.73	36.04	−0.144
cds.evm.model.ctg607.22	*ALDH3*	Plas ^a^, Cyto ^b^	/	481	53.615	7.87	37.83	−0.084
cds.evm.model.ctg64.16	*ALDH5A-2*	Cyto ^a^, Cyto ^b^	/	165	17.484	8.22	28.45	0.214
cds.evm.model.ctg64.15	*ALDH5A-1*	Extr ^a^, Cyto ^b^	/	138	15.230	5.72	43.04	0.137
cds.evm.model.ctg98.26	*ALDH18A1*	Plas ^a^, Mito ^b^	/	490	53.268	5.98	40.49	0.02

Cytoplasm (Cyto); plasma membrane (Plas); mitochondrion (Mito); Extracell (Extr). ^a^: The result predicted by WoLF PSORT; ^b^: Cell-PLoc.

## Data Availability

All the datasets in this study can be provided upon reasonable request.

## Supplementary Materials

The following supporting information can be downloaded at: https://www.mdpi.com/article/10.3390/ani15010064/s1, Figure S1: Multiple sequence alignment diagram.
